# Video calls at end of life are feasible but not enough: A 1‐year intensive care unit experience during the coronavirus disease‐19 pandemic

**DOI:** 10.1111/nicc.12647

**Published:** 2021-05-05

**Authors:** Alessandro Galazzi, Filippo Binda, Simone Gambazza, Maura Lusignani, Giacomo Grasselli, Dario Laquintana

**Affiliations:** ^1^ Healthcare Professions Department Fondazione IRCCS Ca' Granda Ospedale Maggiore Policlinico Milan Italy; ^2^ Health Sciences Department University of Florence Florence Italy; ^3^ Department of Biomedical Sciences for Health University of Milan Milan Italy; ^4^ Department of Pathophysiology and Transplantation University of Milan Milan Italy; ^5^ Department of Anesthesia, Intensive Care and Emergency Fondazione IRCCS Ca' Granda Ospedale Maggiore Policlinico Milan Italy

The coronavirus disease (COVID‐19) pandemic has turned our lives upside down and virtually all care settings have undergone profound transformations worldwide. In a short time, thousands of patients required hospital treatment, many of them in an intensive care unit (ICU).^1^

One of the most critical aspects for patients, their families and health care professionals (HCPs) was the isolation, which was severe, especially at early stages of the pandemic.^2^ The mortality associated with serious infection necessitated public health measures to restrict family visits to hospitals, in addition to the shortage of personal protective equipment.^3^ Abruptly, the long process of opening ICUs to families^4^, ^5^ was terminated, generating an overall sense of loneliness that overwhelmed not only patients but also their families and HCPs.^6^ Thousands of people died without their families being present.^7^ However, patients did not die alone, nurses and physicians were there and cared them until their last breath.^8^

As HCPs living in a technological era, where the digital *medium* is supposed to bring people together and used to help, several hospitals used video calls to bridge the gap between patients and their families. The extensive use of this method of communication, subsequently recommended by the Italian Critical Care Scientific Societies and by the literature,^9^, ^10^ has pushed our ICU team to use video calls, not only as a window to the external world on a daily basis, but also as a chance for caregivers, family members or friends of patients to say goodbye before the withdrawing life‐sustaining treatments or imminent death.^11^

In this commentary, we would like to share our experience using video calls at end of life (EOL) in the ICUs of Fondazione Scientific Institute for Healthcare and Research (IRCCS) Ca' Granda Ospedale Maggiore Policlinico, in Milan, that was designated as a hub in Lombardy region. We analysed the first year of the pandemic from February 2020, the beginning of our COVID‐19 ICU transformation.

By the term EOL‐video call we mean calls made via tablet or smartphone with a camera on, performed in a structured way, that is, after it was made clear to patient's family members that intensive treatments were going to be suspended to leave space only to palliative treatments or when the patient's death was imminent. The EOL‐video call was always proposed by the consultant physician, who provided daily clinical information to the family members for the entire duration of the ICU stay. If the family members consented, the video call was handled by the physician and the nurse in charge. The timing of the EOL‐video call was agreed with the relatives, sometimes engaging chaplaincy support, if desired. Before starting the video call, all the ICU staff were informed, and we attempted to reduce distractions in the ICU environment (alarms silenced, curtains drawn) and the patient was prepared to be seen by family members or relatives, considering that some of the procedures occurred during ICU stay may have changed their appearance.^12^

During this period, 297 patients were admitted to ICUs, and 70 (23.6%) died. Only two patients were conscious at the time of ICU admission. The distribution of EOL‐video calls over time is depicted in Figure 1.

**FIGURE 1:**
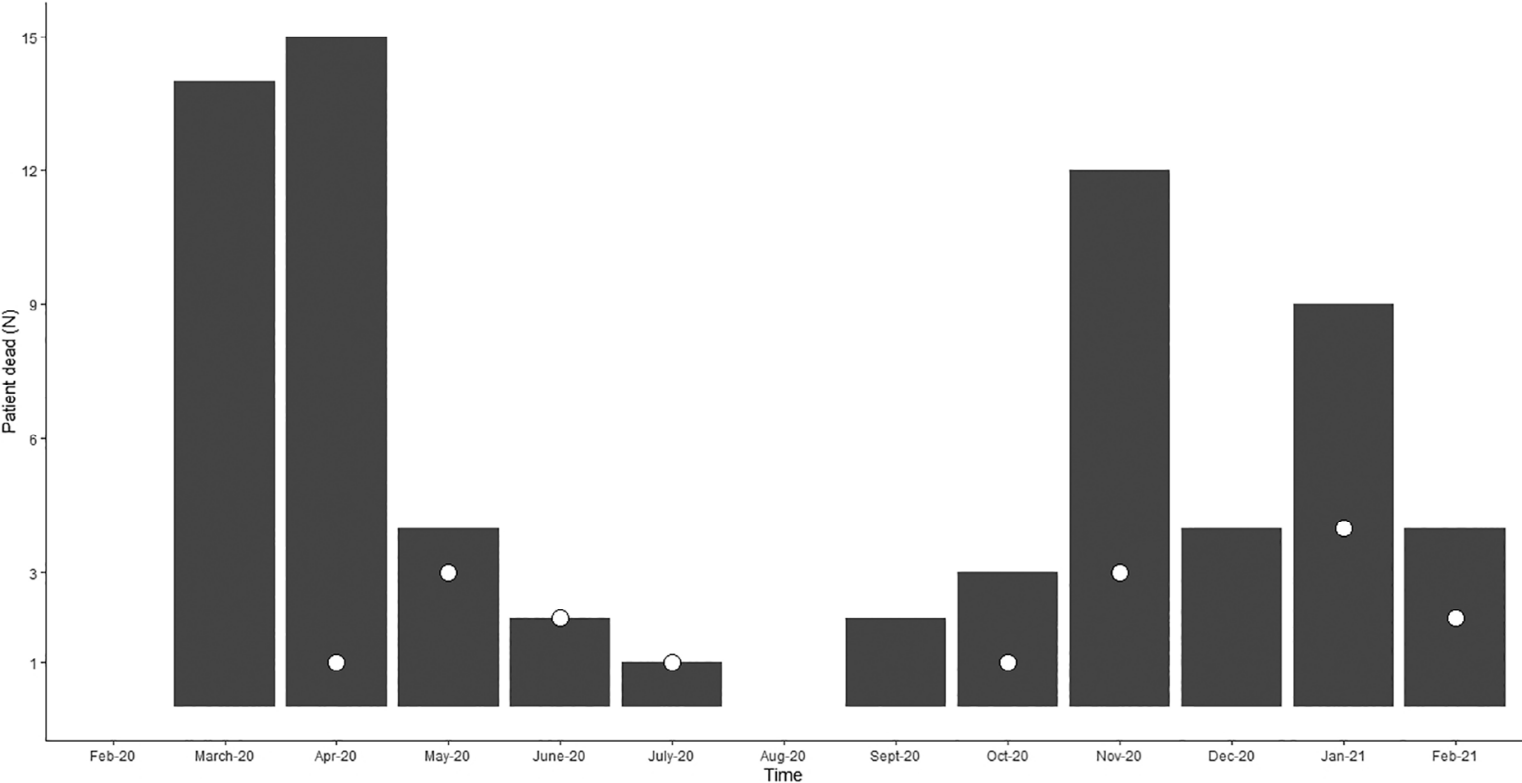
Bars display the number of patients who died during the study period, and the dots represent the number of video calls performed at end of life. Time denotes month‐year

Overall, the pattern describes the first and second COVID‐19 wave in Italy, with deaths peaking during April and November 2020.

From April, we made video calls whenever it was possible. During this time, we assisted family‐members to say goodbye to 17 patients using video calls. Patients were predominantly male (76.5%), without meaningful difference in age (mean difference −1, 95%CI: −5.7 to 7.6 years) between those who were and were not virtually seen by their relatives before death. Out of 53 patients, 15 (28.3%) died with nurses or physicians at their bedside, because relatives did not feel able to go through the process virtually, whereas 38 (71.7%) did not have the suitable technology for video calling. Interestingly, the latter were also younger (mean difference 2.3, 95%CI: −5.3 to 9.9 years). For five patients we could not set up the video calls in time, as death was not expected, and they died while resuscitation procedures were ongoing. 75.7% of patients who were not able to be reached by family members were also characterized by a heterogenous social context. Friends were the only contact available for five patients, whereas two patients had no family or friends. In only one case, was the only contact an uncle. For the majority of patients, their spouse was their outside contact. Despite the absence of a clear pattern in the age distribution among patients' contact, friends were the main contacts of the younger patients, i.e., mean 53.3 (SD 14.5) years.

During each video call, both physicians and nurses remained at the bedside, trying to make family members as comfortable as they could, reassuring the relatives that the patient was comfortable and not in pain, which was the most recurrent concern expressed by relatives. Requests to caress the patient's face or hold their hands were welcomed as well.

The choice of using video calls *peri mortem* was much debated by the ICU team, because some physicians and nurses believed that this modality could be traumatic for family members and for the staff. As recently reported, some ICUs in the United Kingdom also deemed patients ineligible at end of life for video calls.^13^ Indeed, several barriers are identified. Some families may feel that seeing their loved one in a different shape (looking gravely ill and with lines and tubes in situ), rather than a good memory they had before hospitalization, is not the best way for them to see the patient as their last memory. Others feel that more training and assistance by specialist staff, i.e. psychologists, could help staff or families to cope with all the process. Indeed, we should remember that HCPs were already under huge pressure, considering the limited medical resources during the first wave, the heavy workload, the risk of infection and the separation from family and friends for a long time, to name a few.^14^

From the family‐members' perspective, the psychological sequalae of video calling or the missed opportunity to say their last goodbye has not been studied yet. However, what prompted us to continue proposing the videocall at EOL was that some family members expressly requested to be able to see their loved one. Some of them also specified the importance of this opportunity as the time for grieving.

However, this experience revealed the uneven distribution in the access to technology, demonstrating the consequences of the digital divide, that is, that not everyone is in a position to be able to access to care services under any circumstances when delivered through digital means. Among families composed of people over 65 years, only 34% have a broadband connection in Italy.^15^ Many patients who died without their spouses virtually present were in this age category. Public health programmes should focus on this missed opportunity, leveraging the devastating effects of COVID‐19 pandemic to fill this gap.

In conclusion, we believe that video calls at end‐of‐life have been a valuable tool when contextualized in a situation of total isolation, when restrictions or prohibition of family visiting were the biggest barrier to communication and to family‐ and patient‐centred care. The COVID‐19 pandemic has also revealed that a digital divide might have serious consequences on several aspects of our life, including the chance to say goodbye to loved ones. EOL care with family members at the bedside is, and must always be, the first choice.

## AUTHOR CONTRIBUTION

Alessandro Galazzi: Conceptualization, Investigation, Data Curation, Writing—Original Draft. Filippo Binda: Conceptualization, Investigation, Writing—Original Draft. Simone Gambazza: Methodology, Formal Analysis, Writing—Original Draft. Maura Lusignani: Methodology, Writing—Review and Editing. Giacomo Grasselli: Writing—Review and Editing, Supervision. Dario Laquintana: Resources, Writing—Review and Editing, Supervision.
